# CB-Art Interventions Implemented with Mental Health Professionals Working in a Shared War Reality: Transforming Negative Images and Enhancing Coping Resources

**DOI:** 10.3390/ijerph17072287

**Published:** 2020-03-28

**Authors:** Dorit Segal-Engelchin, Netta Achdut, Efrat Huss, Orly Sarid

**Affiliations:** Spitzer Department of Social Work, Ben-Gurion University of the Negev, POB 653, Beer-Sheva 84105, Israel; nettaach@bgu.ac.il (N.A.); ehuss@bgu.ac.il (E.H.); orlysa@bgu.ac.il (O.S.)

**Keywords:** mental health professionals, shared war realty, distress, art-based intervention, war-related stressors, coping resources

## Abstract

Research on mental health professionals (MHPs) exposed to a shared war reality indicates that they are subject to emotional distress, symptoms of posttraumatic stress disorder, and vicarious trauma. This article focuses on a CB-ART (cognitive behavioral and art-based) intervention implemented during the 2014 Gaza conflict with 51 MHPs who shared war-related experiences with their clients. The intervention included drawing pictures related to three topics: (1) war-related stressors, (2) coping resources, and (3) integration of the stressful image and the resources drawing. The major aims of the study were (1) to examine whether significant changes occurred in MHP distress levels after the intervention; (2) to explore the narratives of the three drawing and their compositional characteristics; and (3) to determine which of selected formats of the integrated drawing and compositional transformations of the stressful image are associated with greater distress reduction. Results indicate that MHP distress levels significantly decreased after the intervention. This stress-reducing effect was also reflected in differences between the compositional elements of the ‘stress drawing’ and the ‘integrated drawing,’ which includes elements of resources. Reduced distress accompanied compositional transformations of the stressful image. MHPs can further use the easily implemented intervention described here as a coping tool in other stressful situations.

## 1. Introduction 

Since the onset of the Al-Aqsa Intifida in September of 2000, Israeli society has been witnessing continual terrorist attacks by Hamas and other terrorist organisations, including suicide bombings, drive-by shootings, knife and gun attacks, and missile attacks in urban settings launched from the Gaza strip. Hamas’s ongoing threat against Israeli civilians has led to several military operations. The current study was conducted during Operation “Protective Edge”, also known as the 2014 Gaza conflict. This operation was launched in the summer of 2014 in response to the substantial increase in Hamas’s rocket attacks against Israeli communities, firing on an almost daily basis [1].

During Operation “Protective Edge”, which lasted 50 days, more than 4500 rockets were launched towards Israel from Gaza. This operation and the period immediately preceding it represented an intense period of rocket and mortar fire against Israel’s civilian population. Although the range of these rockets covered more than 70% of Israel’s civilian population, those residing in communities near the Gaza Strip were most affected, having only 15 seconds to seek shelter. During this time, six civilians and 67 soldiers in Israel were killed, more than 1600 civilians were harmed, and an estimated 10,000 civilians evacuated their homes. In the Gaza Strip, approximately 2,125 Palestinians were killed [1]. 

Mental health professionals (MHPs) are among the first responders to address the needs of traumatized people following exposure to large-scale disasters, including terrorist attacks and wars. In the southern region of Israel, which has been subject to missile attacks from Gaza since 2001, MHPs encounter a double exposure to war-related trauma as community members and as professionals providing service to terror victims [2]. This situation in which MHPs are coping with the same traumatic event as their clients is referred to as “shared trauma,” “shared tragedy,” or “shared traumatic reality” [3,4,5]. Shared traumatic situations typically occur in communal disasters, such as natural disasters and war [6]. MHPs working in shared traumatic situations face multiple levels of vulnerability to traumatization, including direct, secondary, and vicarious traumatization [3]. 

The negative consequences of shared reality situations have been well documented. These consequences may include emotional distress during a traumatic event [6], as well as immediately after a traumatic event and up to a year later [5]. Cohen et al. [7], who interviewed therapists working with traumatized children following the shared traumatic reality of the Second Lebanon War, found high levels of anxiety, stress, and symptoms of posttraumatic stress disorder (PTSD) among the therapists. Similar findings emerged from Finklestein et al.’s [8] study of MHPs working in areas affected by repeated rocket attacks from the Gaza Strip, indicating that MHPs were at risk for both PTSD and vicarious trauma (VT) symptoms. Those who lived in the more affected area were at even greater risk for developing PTSD and VT symptoms. Additional studies have shown similar associations among level of exposure to terror attacks, PTSD symptoms, and emotional distress [6,9], providing further support for an incremental dose effect [6]. However, other studies have not found an association between exposure levels and emotional distress [10,11]. Increased levels of PTSD symptoms also have been reported among physicians and nurses exposed to a shared war-related reality in Israel [10,12,13] and in Gaza [14,15]. 

Work under shared reality conditions exposes MHPs to the blurring of boundaries between professional and personal lives [4,16,17], including boundaries between work and family loyalties [5,18]. Research also points to the blurring of boundaries between MHPs and their clients [5], manifested in their difficulty separating their personal experience from that of their clients [19]. 

Several studies on the effects of working in a shared war reality have reported a decrease in perceived professional competence among MHPs [5] and a sense of being deskilled [3]. However, other studies reveal a strong perception of professional competence [19] and high levels of professional confidence [9] among these MHPs.

Work in a shared traumatic reality also has been associated with positive consequences. These consequences may include a sense of growth, both personal and professional [4,10,16,19], and a sense of resilience [19]. Post-traumatic growth also has been reported among nurses working in a shared war–related reality in Israel [10] and in Gaza [15]. Positive consequences also can include heightened intimacy in the therapeutic relationship [4,20], a strong therapeutic alliance [19], a high level of work satisfaction, and a sense of agency and helpfulness [7].

The overall picture that emerges from studies of MHPs exposed to a shared traumatic reality stresses the importance of interventions designed to alleviate their emotional distress, particularly among those operating in areas highly exposed to armed conflicts and terror attacks. Interventions for MHPs in traumatic events mainly consist of group support [21,22], individual or group supervision [7], and debriefing sessions [7,9]. Research findings, however, have called into question the effectiveness of debriefing methods in alleviating symptoms of stress among MHPs and disaster workers [9,23]. One possible explanation for the ineffectiveness of debriefing methods is that MHPs view participation and sharing as integral to the organizational culture of mental health services, rather than as a unique intervention tailored to alleviate their war-related stress [9]. Another plausible explanation is that emotional turmoil and thoughts related to traumatic experiences do not lend themselves to easy verbalization [24]. The inadequacy of conventional verbal methods in the context of disasters points to the need to search for alternative methods of self-care for MHPs in shared war situations. To address this need, Huss, Sarid, and Cwikel [25] developed an art-based intervention model for stress reduction and self-care for social workers operating in a war zone during the Iron Cast Operation (2008). They based this intervention model on the use of a single drawing in a single group session. In the first stage of the intervention, social workers were asked to draw one image of their war experience as social workers. They were then instructed to identify the sources of their stress and their stress reactions within the artwork and to change their artwork by adding sources of coping and resilience. Allowing the social workers to change their artwork helped them to gain a sense of control over diffuse sources of anxiety. Huss and Sarid [26] have found that transformation of stressful visual images through drawing and in the imagination is linked to decreased levels of work-related stress among health care professionals. These findings demonstrate the efficacy of transforming a stressful image without extensive verbalization for stress reduction.

This article focuses on a CB-ART (cognitive behavioral and art-based) intervention for distress reduction that was developed based on these earlier findings. This intervention was implemented with MHPs who shared war-related experiences and distress with their clients during Operation “Protective Edge”. 

### The Current Study

The conceptual framework used in this study was based on the Stress and Coping Model (SCM) [27] and on the “art as therapy” orientation, which highlights the healing qualitaties of art making [28,29,30,31].

The aims of the current study were (1) to examine whether significant changes occurred in distress levels among MHPs at the end of the intervention; (2) to explore the narratives of the three drawing (e.g., stress, resources, and integrated drawings) and their compositional characteristics; (3) to determine which compositional elements of the stressful image that were transformed within the ‘integrated drawing’ were associated with greater distress reduction; and (4) to determine which of the selected formats for the ‘integrated drawing’ (e.g., a new sheet of paper, ‘stress drawing,’ or ‘resources drawing’) were associated with greater distress reduction. 

## 2. Methods

Three CB-ART workshops were implemented with MHPs in southern Israel during the 2014 Gaza conflict at the social work department in Ben-Gurion University of the Negev. MHPs were recruited through advertisements on the university website, emails sent after the war began to health and social service agencies in the community, and through a snowball sampling technique. All participants were employed in health or social services agencies and were both working and living in the war zone. 

Before the workshop began, the authors described the objective and procedure of the study and emphasized that participation in the workshop did not require participation in the study. All MHPs who participated in the workshops chose to take part in the study. They were asked to note their level of distress at the beginning and end of the workshop. At the end of the workshop, they also were asked to note the compositional elements that they used in the ‘stress drawing’ and to describe the transformations that they had made in the compositional elements of the stressful image within the ‘integrated drawing.’ To enable pre–post comparisons on an individual level, MHPs were asked to provide the last four digits of their national ID number on both the questionnaire and the three drawings. They were told that this information was needed for statistical purposes and would not be used to identify them.

The research was approved by the departmental ethics committee at Ben-Gurion University of the Negev. All participants signed consent forms agreeing to have their drawings and questionnaire used in research. 

### 2.1. CB-Art Intervention Description 

The two-hour workshop started with a short introductory lecture on stress responses to disasters and the debilitating effect of a negative distressing image, symptom, or memory on negative mood states. We explained how drawings can be analyzed by both the narrative attached to them and their compositional elements, such as shape, size, colors, and placement of the images on the paper [32,33]. 

In the first phase of the intervention, participants were asked to draw their current emotions and thoughts relating to the war situation (referred to here as the *stress drawing*). They were then asked to write a short description of their artwork on the back of their drawing. Participants presented their drawings within the group setting, describing what they had drawn, and shared their narrative. The group then discussed the compositional characteristics of each drawing. The aim of this phase was to enable MHPs to identify the sources of their war-related stress as well as the compositional elements that characterized their stressful image. 

After drawing about their current condition, participants were asked to draw a new picture that reflected on their personal and social resources that could enable them to better cope with stressful situations (referred to here as the *resources drawing*). Again, they were asked to write a short description of their artwork on the back of it. Drawings were shared within the group setting and their compositional elements noted. The aim of this phase was to enable MHPs to identify coping resources at their disposal as well as the compositional elements that characterized their resources drawing.

In the last phase of the intervention, participants were asked to draw a picture that integrated the stressful image and the resources drawing (referred to here as the *integrated drawing*). Participants had the option to draw a new picture or to add elements to either the stress drawing or the resources drawing. The integrated drawings were shared within the group setting and their compositional elements discussed and compared to those of the stress and resources drawing. The purpose of the integrated drawing was to enable participants to learn how to “build bridges” between their resources and their distress image, symptom, or memory. The drawings in all three phases described above were created on A-4 paper with oil pastels. 

### 2.2. Sample

To ensure the homogeneity of the sample, participants in the three workshops were compared by their demographic variables. Chi-square tests revealed no significant differences in gender, marital status, country of birth, education level, religion, degree of religious observance, or perceived financial situation. For this reason, we pooled the three groups into one. Table 1 presents the demographic characteristics of the pooled sample. The average age was 37 years, and most were female (86%) and Israeli born (82% ), were married or lived with a partner (63%), and had children (52%). Almost all participants were Jewish (96%), and most defined themselves as secular (72%). About half of the participants (52% ) viewed their financial situation as fair.

Participants included: 45 (88.2%) social workers, 5 (9.8%) psychologists and one psychiatrist (1.9%). All participants drew the three drawings included in the CB-ART intervention described above. With regard to the selected format for the integrated drawing, 21 participants chose to draw a new picture; 3 participants chose to add elements to the stress drawing; and 27 participants chose to add elements to the resources drawing.

### 2.3. Measures

#### 2.3.1. Distress Level

Participant distress level was measured using the Subjective Units of Distress Scale (SUDS) [34]. Respondents were asked to assess their level of distress on an 11-point scale ranging from 0 (absence of distress) to 10 (extreme level of distress). The SUDS has been used in previous studies that evaluated the efficacy of art-based interventions in reducing stress [26,35].

#### 2.3.2. Compositional Elements 

We used a compositional element scale, based on the compositional analysis of image transformation [26,36], to examine the compositional elements of the stressful image and its transformed elements within the integrated drawing. This scale covered five compositional elements: object, color, placement, size, and background. Participants were asked to fill in this scale for both the stress drawing and the integrative drawing. 

#### 2.3.3. Statistical Analyses 

To investigate whether significant changes occurred in participant distress levels following the intervention, in a first step, we employed a paired sample *t*-test to compare pre–post SUDS scores. In a second step, we used descriptive statistics to analyze the compositional elements of the three drawings. Statistical tests for examining differences in these characteristics were not carried out because of the constraint of small cells. In a third step, we used independent sample *t*-tests to examine whether transformations in the compositional elements of the stress drawing within the integrated drawing were related to a greater reduction in SUDS scores. For this purpose, we computed a variable based on the difference between the SUDS score at (t2) and the SUDS score at (t1) (referred to here as *SUDS-difference score*). We then examined three types of transformations within the integrated drawing: (1) object transformation (e.g., addition versus change in or omission of objects), (2) color transformation (e.g., number and types of colors used), and (3) size transformation (e.g., reduction versus non-reduction of the stressful image size). 

An independent samples *t*-test was also used to examine whether the selected format for the integrated drawing (e.g., a new sheet of paper, addition of elements to the stress drawing, or addition of elements to the resources drawing) was related to a greater reduction in the SUDs scores. Because only three participants drew the integrated drawing on the resources drawing, we created two categories: the first comprised those who added elements from the resources drawing to the stress drawing, whereas the second comprised participants who either drew a new picture or added elements from the stress drawing to their resources drawing. 

## 3. Results 

### 3.1. Pre–Post SUDS Scores 

Table 2 presents the results of the paired sample *t*-tests conducted to examine differences in MHP SUDS scores between the beginning and end of the intervention. As Table 2 shows, the scores significantly decreased after the intervention, suggesting efficacy of the process. The mean difference was 1.51 (on a 0–10 scale).

### 3.2. Compositional Characteristics of the Mhps’ Drawings and Their Explanatory Narratives

The second aim of the study was to explore the compositional characteristics of the three drawings and their explanatory narratives. Here we present the descriptive statistics of the compositional characteristics of the three drawings, followed by two illustrative examples of drawings and explanatory narratives. 

As Table 3 shows, there were substantial differences among the three drawings in the compositional elements. Almost half of the stress drawings (49%) had no background, compared to only a little more than a third of the resources drawings (37.3%), and less than a third of the integrative drawings (29.4%). A quarter of the stress drawings (25.5%) were composed of a single object, and in 37.3% of the drawings, the stressful image was placed at the center. In contrast, less than 10% of the resources drawings (7.8%) and of the integrative drawings (9.8%) were composed of a single object, and a considerably smaller percentage of these drawings had their image placed at the center of the drawing (19.6% and 13.7%, respectively). More than half of the stress drawings (54.9%) and of the resources drawings (54.9%) consisted of medium and large objects, compared to 45.1% of the integrated drawings. Additionally, in almost half of the stressed drawings (47.1%), black emerged as the dominant or only color, compared to only 3.9% of the resources drawings and 21.6% of the integrated drawings. A similar pattern was observed with regard to the dominant use of grey within the stress drawings (11.8%), compared to its dominant use within the resources drawings (2.0%) and the integrated drawings (5.9%). In contrast, in about a third of the resources drawings and the integrated drawings, green emerged as the dominant or only color, compared to only 7.8% of the stress drawings.

#### Illustrative Examples of the Drawings 

In this section, we present two illustrative examples of these drawings and explanatory narratives: one by an MHP who selected a new sheet of paper as the format for her integrative drawing, and the other by an MHP who selected the stress drawing as the format for his integrative drawing.

**Figure 1:**
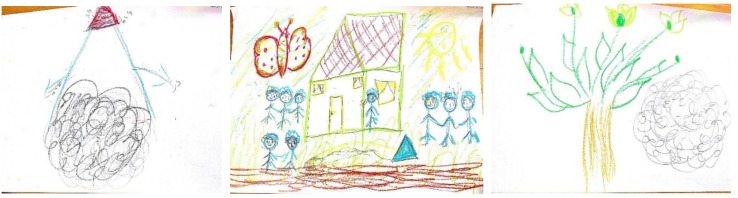
Selecting a new sheet of paper as the format for the integrative drawing.

With regard to the stress drawing in Example 1 (drawing at far left in Figure 1), the MHP explained her drawing as follows: “I drew the emotional turmoil that I have been experiencing because of my concern over the safety of my family, including my children and grandchildren, family friends who are in active duty military service during the war, and my clients and staff members”. These three groups of people are displayed in the three vertexes of the triangle that appears in the drawing, with the clients and staff members placed at the upper vertex, as noted in the words added to her drawing. With regard to the resources drawing (drawing at the center in Figure 1), the MHP noted the following: “I drew my home and family members, which I view as a major resource enabling me to better cope in stressful situations”. With regard to the integrative drawing (drawing at the right in Figure 1), the MHP stated that “The drawing expresses my lessened feelings of emotional turmoil”. 

Analysis of the compositional elements of her three drawings reveals substantial differences among them. As can be seen, the stressful image was represented by a single, primarily black, large object, placed at the center of the drawing, with no background. In contrast, the resources and the stress drawings are characterized by the use of several mixed-sized objects and lighter, “optimistic” colors. In terms of the transformed compositional elements of the stressful image within the integrated drawing, the MHP explained that “I had omitted the triangle representing the sources of my emotional turmoil and decreased the size of the stressful image within the integrated drawing”. One can also see that she has moved the stressful image from the center of the page. This compositional transformation appears as an additional indicator of her lessened feelings of distress.

**Figure 2:**
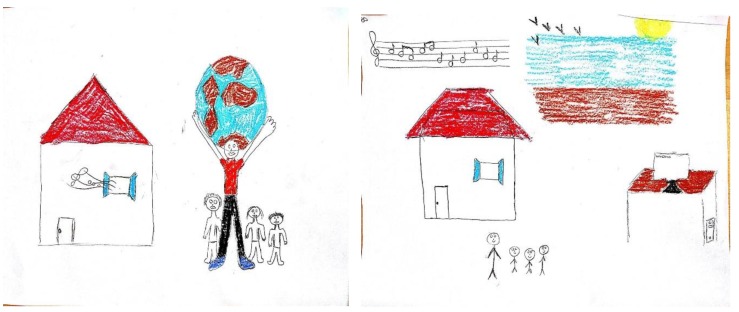
Selecting the stress drawing as the format for the integrative drawing.

The stress drawing in Example 2 (left in Figure 2) initially included only the image of a man holding a huge ball. The MHP described his drawing as follows: “In this drawing the focus is on myself. The huge ball symbolizes the extreme stress and burden that I have been encountering during the war in multiple domains due to the need to address my family and clients’ needs simultaneously”. With regard to the resources drawing (right, Figure 2), he noted that “I drew the resources that usually help me calm down in stressful times: my home, family, music, and the beach”. With regard to the integrative drawing (right, Figure 2), the MHP stated that “I have added my family and home to the stress drawing because they help me to cope with any problem or stressful situation that I encounter”.

Analysis of the compositional elements of the drawings in Example 2 also reveals substantial differences between the drawings. Whereas the initial stressful image was represented by a single large object with no background, the resources drawing is characterized by the use of several mixed-sized objects, scattered all over the paper. With regard to the integrated drawing, the addition of objects to the stress drawing enabled the MHP to alter the proportion of the stressful image within the drawing, as well as to situate it within his everyday social context. These compositional transformations reflected the decrease in his feelings of distress. 

The two examples of drawings and themes emerging from the explanatory narratives presented above, which were evident in the explanatory narratives of the vast majority of participants, agree with the overall picture emerging from the descriptive statistics of the compositional characteristics of the three drawings displayed in Table 3. 

### 3.3. SUDS-Difference Scores by the Selected Format of the Integrated Drawing and Compositional Transformations

In the current study, we also sought to determine which of selected formats and the transformed compositional elements of the stressful image within the integrated drawing are associated with greater SUDS reduction. Table 4 presents the SUDS-difference scores by the selected format for the integrated drawing and transformed compositional elements. As shown in Table 4, we found statistically significant differences with regard to size transformation within the integrated drawing and to the selected format of the integrated drawing. Participants who reduced the initial size of the stressful image within the integrated drawing had a greater SUDS-difference score (−1.87 [1.22]) than those who maintained the initial size of the stressful image (−1.13 [1.48]). Additionally, participants who drew the integrated drawing on either the resources drawing or on a new sheet of paper had a higher SUDS-difference score (−1.73 [0.15]) than participants who drew the integrated drawing on the stress drawing (−0.92 [0.86]).

## 4. Discussion

This study focused on a CB-ART intervention implemented with MHPs who shared war-related experiences and distress with their clients during the 2014 Gaza conflict. Results indicate that MHPs’ levels of distress significantly decreased after the intervention, suggesting its efficacy. Further evidence of the efficacy of a CB-ART intervention in reducing disaster-related distress is derived from Segal-Engelchin et al.’s [35] study of Nepalese students living in Israel during the 2015 Nepal earthquake, who were indirectly exposed to the disaster that struck their country. A plausible explanation for the decline in MHPs’ levels of distress at the completion of the intervention may be that drawing and identifying the war-related stressors as well as personal and social resources increased their sense of control in the war situation. Transforming the compositional elements of the stress drawing within the integrated drawing may have also enhanced MHPs’ sense of control. Previous studies on art-based interventions suggest that the active management of a stressful image leads to an enhanced sense of control [25,26]. Additionally, it may be that modifying the compositional elements of the stress image within the integrated drawing allowed the MHPs to modify its emotional content into a more enabling meaning [32]. This possibility is reflected in the explanatory narrative ascribed by the MHP in Example 1 to her integrative drawing, in which the image of war-related stress was modified, indicating that the drawing mirrored the lessened feelings of emotional turmoil. Another possible explanation lies in the integrated drawing, in which the stressful image and the coping resources were simultaneously displayed. This depiction may have enabled the MHPs to view war-related stressors and coping resources at their disposal as two interrelated entities of their war experience. This, in turn, may have led them to perceive the war-related stressors as less threatening and more manageable. The use of arts as a tool that enhances manageability has been reported by Huss and Samson [37] in their study of a group of recovering cancer patients.

One aim of the study was to explore participants’ narratives of the three drawings (e.g., stress, resources, and integrated drawings) and their compositional characteristics. Their narratives of the stressful image, as demonstrated in the above two examples, reflected their emotional turmoil, feelings of extreme distress, and concern about the safety of their loved ones and their clients. A further prominent theme in their narratives, which was also evident in the narratives of the other participants, was the emotional burden that stemmed from assisting their clients while coping with their own anxieties and caring for their family members. These narratives corroborate findings of previous studies indicating high levels of anxiety and emotional distress among MHPs in shared trauma situations [6,7,8,9] as well as the blurring of boundaries between work and family loyalties [5,18,25].

Two major resources that enabled better coping with stressful situations emerged from the narratives of the resources drawing. One was the family and home environment, and the other was related to social-leisure activities, such as listening to music and going to the beach. Previous studies indeed indicate that family is an essential source of support for MHPs in shared war realities [5]. Their narratives of the integrated drawing reflect lessened feelings of emotional turmoil as well as a perceived ability to better cope with the shared war reality. 

The different narratives ascribed to the three drawings are also expressed in the different compositional characteristics of these drawings. Our quantitative findings revealed substantial differences among the three drawings in the compositional elements, as illustrated in the two examples given. The stress drawings were generally characterized by a single, predominantly black, large–medium-sized object, placed at the center of the drawing, with no background. In contrast, both the resources and integrative drawings were typically characterized by the use of several mixed-sized objects and lighter optimistic colors, scattered all over the drawing. The use of single large objects and intense black lines to depict war-related stressors have been reported previously [25], and comparable compositional characteristics of stressful images also have been found in a study of children with cancer [38]. The use of the color black is associated with stress and depression in the diagnostic art therapy literature [39,40,41,42]. While diagnostic analyses of color is based on universal measures, other art therapy directions point to the cultural significance of specific colors [43]. On an Israeli cultural level, the color black is also associated with negative experiences such as mourning, a situation in which people wear black clothes, and negative moods, which are described as “black”. 

An additional objective of the current study was to determine which of the transformed compositional elements of the stressful image within the integrated drawing was associated with greater SUDS score reduction. The results indicate that size transformation of the stressful image within the integrated drawing was the only transformed compositional element significantly associated with greater SUDS score reduction. This finding can be interpreted in two ways. MHPs who experienced greater distress reduction after identification of their war-related stressors and coping resources in the framework of the first two phases of the intervention tended to decrease the size of the stressful image within the integrative drawings. It could also be, however, that the greater distress reduction resulted from the size transformation of the stressful image rather than being the cause of the size transformation. It is possible that the size modification of the stressful image and its proportion relative to the objects symbolizing various coping resources enabled MHPs to feel an increased sense of control and competence, resulting in their greater SUDS score reduction. Further research is needed to determine the direction of causality between SUDS score reduction and size transformation of the stressful image.

Of interest, we found an association of selecting the resources drawing or a new sheet of paper for the integrated drawing with significantly greater SUDS score reduction. This finding suggests that in the process of transforming the stressful image, attention should also be given to the context where the stressful image is placed, in addition to the transformed compositional elements. The placement of the stressful image in a new context that encompasses MHPs’ personal and social resources may lessen its threatening features, resulting in an enhanced sense of agency. It is also possible that the selection of a new context for the stressful image is an indication of reduced levels of distress. Further investigation may shed light on the causal relationship between the format selected for the integrative drawing and SUDS score reduction.

Several limitations of the current study should be acknowledged. The first is related to the cross-sectional design that does not allow for determination of the long-term impact of the CB-ART intervention. Follow-up studies are needed to investigate the long-term effectiveness of this intervention. The second limitation is related to the lack of a control group. This limitation is inherent to this type of quick-response intervention in times of disaster, where the evaluative research must be conducted rapidly and with regard for the well-being of all the people involved. A third limitation is the relatively small sample size, which precluded rigorous statistical tests to examine the differences among the various compositional elements of the three drawings. Future studies using larger samples may enrich our understanding of the role that compositional elements play in shaping participants’ perception of stressful images, resource images, and integrated images. Further research is also needed to examine the contribution of the different components of the intervention (e.g., each of the three different drawings and the group setting) to stress reduction. 

## 5. Conclusions 

The current study marks the first attempt to examine the effectiveness of a CB-ART intervention implemented with MHPs under actual wartime conditions. In this study, the concepts presented in the Stress and Coping Model [SCM, 27] served as a framework to examine the ways that a CB-ART intervention, based on drawing, can help MHPs to express their stress as well as to acknoewledge their coping strategies, and integrate these two elements in the context of war. Within the drawing process, the identification of the compositional elements of the three drawings and the creation of manipulations within the integrated drawing enabled participants to actively appraise which compositional transformations decreased their distress and enhanced their adjustment and coping [44,45]. Our findings suggest that participants’ conscious cognitive processing of the compositional transformations altered their interpretation of the stressful images, which in turn decreased their distress levels.

The study findings make an initial contribution to understanding the ways that stressful images and resource images are integrated on the paper using compositional transformations, as well as the ways in which this process assists in reducing MHP distress levels when they are operating in a shared war reality. The findings add to the art therapy literature on positive psychology and on the healing qualitaties of art making as in “ art as therapy” orientations [28,29,30,31]. 

On a practical level, this study offers an easily implemented tool for distress reduction among MHPs in shared trauma situations. The CB-ART intervention provided MHPs an opportunity to depict their war-related stressors as well as their coping resources on the page and to discuss both images and access new thoughts and understanding regarding ways to manage stress in extremely stressful situations. As such, the CB-ART intervention not only may have enriched their coping resources but also have become a coping resource in itself, which they can use in traumatic situations in the future as a self-care strategy. 

## Figures and Tables

**Table 1:** Demographic characteristics of study participants (*N* = 51).

Characteristics	*n*	% or Mean (SD)
Age		37.5 (12.5)
23–35	26	52.0
36–54	19	38.0
55 and above	5	10.0
Gender		
Male	7	14.0
Female	43	86.0
Marital status		
Single	16	32.0
Married	23	46.0
Cohabiting	9	18.0
Divorced/Separated	2	4.0
Has children		
Yes	26	52.0
No	24	48.0
Country of birth		
Israel	41	82.0
Other (US, Europe, FSU)	9	18.0
Religion		
Jewish	49	96.0
Muslim	2	4.0
Degree of religiosity		
Secular	36	72.0
Traditional or religious	14	28.0
Education		
Academic-B.A. degree	36	72.0
Academic-M.A. degree	14	28.0
Perceived financial situation		
Bad	4	8.0
Fair	26	52.0
Good or very good	20	40.0

**Table 2:** Pre–post Subjective Units of Distress Scale (SUDs) scores (*N* = 51).

	SUDS Score Mean (SD)	Paired Sample *t*-Test
SUDS score at t1	5.97 (2.22)	
SUDS score at t2	4.46 (1.98)	
Mean difference	1.51 (1.39)	7.41 **

** *p* < 0.01.

**Table 3:** Descriptive statistics of the compositional characteristics of the three drawings (*N* = 51).

	Drawings		
	Stress Drawing (%)	Resources Drawing (%)	Integrated Drawing (%)
Background			
No background	49.0	37.3	29.4
Had background	51.0	62.7	76.6
Number of objects			
One	25.5	7.8	9.8
Several	74.5	92.2	90.2
Object placement			
Center of the drawing	37.3	19.6	13.7
All over the drawing	62.7	80.4	86.3
Object size			
Large-medium	54.9	54.9	45.1
Small or mixed	45.1	45.1	54.9
Colors			
Black			
None or minor	52.9	96.1	78.4
Dominant or only	47.1	3.9	21.6
Green			
None or minor	92.2	68.6	66.7
Dominant or only	7.8	31.4	33.3
Grey			
None or minor	88.2	98.0	94.1
Dominant or only	11.8	2.0	5.9

**Table 4:** SUDS-difference scores by the selected format of the integrated drawing and compositional transformations: stress-drawing versus integrated drawing (*N* = 51).

	SUDS-Difference Mean (SD)	Independent Sample *t*-Test
Selected format for the integrative drawing		1.828 *
Stress drawing	−0.92 (0.86)	
Resources drawing or a new drawing	−1.73 (0.15)	
Shape transformation		0.150
Addition of shapes	−1.50 (1.40)	
Change or omission of shapes	−1.60 (1.51)	
Color transformation		−0.78
Yes	−1.59 (1.36)	
No	−1.20 (1.54)	
Size transformation		−1.786 *
Reduction of initial size	−1.87 (1.22)	
Maintenance of initial size	−1.13 (1.48)	

* *p* < 0.05.

## References

[1] Israel Ministry of Foreign Affairs (2015). The 2014 Gaza Conflict: Factual and Legal Aspects. Jerusalem: Ministry of Foreign Affairs. https://mfa.gov.il/MFA/ForeignPolicy/IsraelGaza2014/Pages/2014-Gaza-Conflict-Factual-and-Legal-Aspects.aspx.

[2] Baum N. (2014). Professionals’ double exposure in the shared traumatic reality of wartime: Contributions to professional growth and stress. Br. J. Soc. Work.

[3] Saakvitne K. (2002). Shared trauma: The therapists’ increased vulnerability. Psychoanal. Dialogues.

[4] Tosone C., Nuttman-Shwartz O., Stephens T. (2012). Shared trauma: When the professional is personal. Clin. Soc. Work J..

[5] Dekel R., Baum N. (2010). Intervention in a shared traumatic reality: A new challenge for social workers. Br. J. Soc. Work.

[6] Freedman S.A., Mashiach R.T. (2018). Shared trauma reality in war: Mental health therapists’ experience. PLoS ONE.

[7] Cohen E., Roer-Strier D., Menachem M., Fingher-Amitai S., Israeli N. (2014). “Common-Fate”: Therapists’ benefits and perils in conducting child therapy following the shared traumatic reality of war. Clin. Soc. Work J..

[8] Finklestein M., Stein E., Greene T., Bronstein I., Solomon Z. (2015). Posttraumatic stress disorder and vicarious trauma in mental health professionals. Health Soc. Work.

[9] Dekel R., Hantman S., Ginzburg K., Solomon Z. (2007). The cost of caring? Social workers in hospitals confront ongoing terrorism. Br. J. Soc. Work.

[10] Lev-Wiesel R., Goldblat H., Eisikovits Z., Admi H. (2009). Growth in the shadow of war: The case of social workers and nurses working in a shared war reality. Br. J. Soc. Work.

[11] Pruginin I., Segal-Engelchin D., Isralowitz R., Reznik A. (2016). Shared war reality effects on the professional quality of life of mental health professionals. Isr. J. Health Policy Res..

[12] Ben-Ezra M., Palgi Y., Wolf J.J., Shrira A. (2011). Psychiatric symptoms and psychosocial functioning among hospital personnel during the Gaza War: A repeated cross-sectional study. Psychiatry Res..

[13] Ben-Ezra M., Palgi Y., Wolf J.J., Shrira A., Hamama-Raz Y. (2013). Somatization and psychiatric symptoms among hospital nurses exposed to war stressors. Isr J. Psychiatry Relat Sci..

[14] Abu-El-Noor N.I., Aljeesh Y.I., Radwan A.S., Abu-El-Noor M.K., Qddura I.A.I., Khadoura K.J., Alnawajha S.K. (2016). Post-Traumatic Stress Disorder among health care providers following the Israeli attacks against Gaza Strip in 2014: A call for immediate policy actions. Arch. Psychiatr. Nurs..

[15] Shamia N.A., Thabet A.A.M., Vostanis P. (2015). Exposure to war traumatic experiences, Post-Traumatic Stress Disorder and post-traumatic growth among nurses in Gaza. J. Psychiatr. Ment. Health Nurs..

[16] Shamai M., Ron P. (2009). Helping direct and indirect victims of national terror: Experiences of Israeli social workers. Qual Health Res..

[17] Somer E., Buchbinder E., Peled-Avram M., Ben-Yizhack Y. (2004). The stress and coping of Israeli emergency room social workers following terrorist attacks. Qual. Health Res..

[18] Dekel R., Nuttman-Shwartz O., Lavi T. (2016). Shared traumatic reality and boundary theory: How mental health professionals cope with the home/work conflict during continuous security threats. J. Couple Relatsh. Ther..

[19] Lavi T., Nuttman-Shwartz O., Dekel R. (2015). Therapeutic intervention in a continuous shared traumatic reality: An example from the Israeli–Palestinian conflict. Br. J. Soc. Work.

[20] Tosone C. (2006). Therapeutic intimacy: A post-9/11 perspective. Smith Coll. Stud. Soc. Work.

[21] Mitchell J.T., Everly G.S., Raphael B., Wilson J.P. (2000). Critical incident stress management and critical incident stress debriefings: Evolutions, effects and outcomes. Psychological Debriefing: Theory, Practice and Evidence.

[22] Wilson J., Sigman M., Raphael B., Wilson J.P. (2000). Theoretical perspectives of traumatic stress. Psychological Debriefing: Theory, Practice and Evidence.

[23] Kenardy J.A., Webster R.A., Lewin T.J., Carr V.J., Hazell P.L., Carter G.L. (1996). Stress debriefing and patterns of recovery following a natural disaster. J. Trauma. Stress.

[24] Huss E., Hafford-Letchfield T. (2019). Using art to illuminate social workers’ stress. J. Soc. Work.

[25] Huss E., Sarid O., Cwikel J. (2010). Using art as a self-regulating tool in a war situation: A model for social workers. Health Soc. Work.

[26] Huss E., Sarid O. (2014). Visually transforming artwork and guided imagery as a way to reduce work related stress: A quantitative pilot study. Arts Psychother..

[27] Lazarus R.S., Folkman S. (1984). Stress, Appraisal, and Coping.

[28] Rosal M., Rubin J. (2001). Cognitive Behavioral Art Therapy. Approaches to Art Therapy: Theory and Technique.

[29] Rubin J. (2016). Introduction. Approaches to Art Therapy: Theory and Technique.

[30] Simon M., Graham A. (2005). Self-Healing Through Visual and Verbal Art Therapy.

[31] Wilkinson R.A., Chilton G. (2013). Positive art therapy: Linking positive psychology to art therapy theory, practice, and research. J. Am. Art Ther. Assoc..

[32] Sarid O., Huss E. (2011). Image formation and image transformation. Arts Psychother..

[33] Sarid O., Cwikel J., Czamanski-Cohen J., Huss E. (2017). Treating women with perinatal mood and anxiety disorders (PMADs) with a hybrid cognitive behavioral and art therapy treatment (CB-ART). Arch. Women’s Ment. Health.

[34] Wolpe J. (1969). The Practice of Behavior Therapy.

[35] Segal-Engelchin D., Sarid O. (2016). Brief intervention effectiveness on stress among Nepalese people indirectly exposed to the Nepal earthquake. Int. J. Ment. Health Addict..

[36] Sarid O., Huss E. (2010). Trauma and acute stress disorder: A comparison between cognitive behavioral intervention and art therapy. Arts Psychother..

[37] Huss E., Samson T. (2018). Drawing on the arts to enhance salutogenic coping with health-related stress and loss. Front. Psychol..

[38] Rollins J.A. (2005). Tell me about it: Drawing as a communication tool for children with cancer. J. Pediatric Oncol. Nurs..

[39] Edwards M., Rubin J. (2001). Jungian Analytic Art Therapy. Approaches to Art Therapy: Theory and Technique.

[40] Goodnow J. (1997). Children’s Drawings.

[41] Khellog R. (1993). Analyzing Childrens Art.

[42] Wadeson H. (2002). The anti-assessment devils advocate. J. Am. Art Ther. Assoc..

[43] Kaplan F. (2000). Now and future ethno-cultural issues. J. Am. Art Ther. Soc..

[44] Ntoumanis N., Edmunds J., Duda J.L. (2009). Understanding the coping process from a self-determination theory perspective. Br. J. Health Psychol..

[45] Tedeschi R.G., Calhoun L.G. (2004). Posttraumatic growth: Conceptual foundations and empirical evidence. Psychol. Inq..

